# Total Saponin from Root of *Actinidia valvata* Dunn Inhibits Hepatoma 22 Growth and Metastasis *In Vivo* by Suppression Angiogenesis

**DOI:** 10.1155/2012/432814

**Published:** 2012-08-22

**Authors:** Guo-Yin Zheng, Hai-Liang Xin, Yan-Fen Xu, Bai Li, Xiao-Feng Zhai, Yuan-Hui Zhang, Chang-Quan Ling

**Affiliations:** Department of Traditional Chinese Medicine, Changhai Hospital, Second Military Medical University, 168 Changhai Road, Shanghai 200433, China

## Abstract

The root of *Actinidia valvata* dunn has been widely used in the treatment of hepatocellular carcinoma (HCC), proved to be beneficial for a longer and better life in China. In present work, total saponin from root of *Actinidia valvata* Dunn (TSAVD) was extracted, and its effects on hepatoma H22-based mouse *in vivo* were observed. Primarily transplanted hypodermal hepatoma H22-based mice were used to observe TSAVD effect on tumor growth. The microvessel density (MVD), vascular endothelial growth factor (VEGF), basic fibroblast growth factor (bFGF) are characterized factors of angiogenesis, which were compared between TSAVD-treated and control groups. Antimetastasis effect on experimental pulmonary metastasis hepatoma mice was also observed in the study. The results demonstrated that TSAVD can effectively inhibit HCC growth and metastasis *in vivo*, inhibit the formation of microvessel, downregulate expressions of VEGF and bFGF, and retrain angiogenesis of hepatoma 22 which could be one of the reasons.

## 1. Introduction

Hepatocellular carcinoma (HCC) is one of the most common malignancies in the world, also the leading cause of cancer-related deaths in China [1]. The prognosis of patients with this type of cancer is primarily determined by the incidence of recurrence after surgery and the occurrence of invading metastases into the remaining liver parenchyma. New metastatic nodules of HCC were supplied by plentiful blood. Therapeutic angiogenesis describes an emerging field of antimetastasis medicine of HCC whereby new blood vessel growth is induced to supply oxygen and nutrients to tumor cells. The growth of this field has exploded in the past decade as a result of the development of recombinant growth factors, the best characterized are the soluble mediators basic fibroblast growth factor (bFGF) and vascular endothelial growth factor (VEGF). Both of these factors stimulate *in vivo* angiogenesis [2, 3].

*Actinidia valvata* Dunn is a shrub mainly growing in eastern China, the roots of which have been widely used for hepatoma treatment for a long time. Continuous studies mainly on its antitumor effects and active ingredients have been carried out in our laboratory for many years. Previous studies have confirmed that injections of *Actinidia valvata *Dunn were proved to restrain the growth of primary hypodermal tumor with no cell-mediate immunity inhibitive effects *in vivo* [4–6]. TSAVD were extracted from the root of *Actinidia valvata* Dunn, which was proved to prevent the metastasis of human hepatocellular carcinoma cells *in vitro* in poir experiments [7], so this study expands on work by observing its effects on hypotransplanted and experimental pulmonary metastasis hepatoma H22 based mouse *in vivo*.

## 2. Materials and Methods

### 2.1. Plant Material 

The roots of *Actinidia valvata* dunn were collected in Changshan county, Zhejiang province, China, in October 2006, and identified by Professor Han-Chen Zheng. Department of Pharmacognosy, School of Pharmacy, Second Military Medical University (SMMU). A voucher specimen (no. 20061005) was deposited in the same department.

### 2.2. Extraction and Isolation of TSAVD 

**Figure 1:**
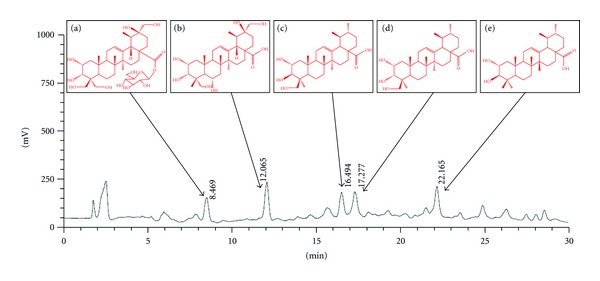
HPLC chromatogram of TSAVD. The major peaks were identified by comparison with standard compounds. Note: (a) 2*α*, 3*β*, 20*β*, 23, 24, 30-hexahydroxyurs-12-en-28-oic acid O-*β*-D-glucopyranosyl ester. (b) 2*α*, 3*α*, 6*α*, 20*α*, 24, 30-hexahydroxyurs-12-en-28-oic acid; (c) 2*α*, 3*β*, 24-trihydroxyurs-12-en-28-oic acid. (d) 2*α*, 3*α*, 24-trihydroxyurs-12-en-28-oic acid. (e) corosolic acid.

The powdered plant material of roots of *A. valvata *was refluxed with 8 times of 80% alcohol solution for 3 times, 1.5 hours each time. The extracting solution was concentrated under reduced pressure twice with 1.5 hours each time to brown syrup, and 1700 mL brown residue was obtained. Centrifugal method of 5000 rpm which lasted 4 min was used to obtain 1400 mL supernatant, which was absorbed by D101 macroporous resin column subsequently, eluted with 5000 mL 80% ethanol to yield major fractions, which were later concentrated under reduced pressure. Finally, the juice was exsiccated and crushed into powder. The HPLC analysis indicated that there were triterpenoids in the TSAVD and the major peaks were identified by comparison with standard compounds and literature, and they were determined, respectively, as 2*α*, 3*β*, 20*β*, 23, 24, 30-hexahydroxyurs-12-en-28-oic acid O-*β*-D-glucopyranosyl ester (a), 2*α*, 3*α*, 6*α*, 20*α*, 24, 30-hexahydroxyurs-12-en-28-oic acid (b), 2*α*, 3*β*, 24-trihydroxyurs-12-en-28-oic acid (c), 2*α*, 3*α*, 24-trihydroxyurs-12-en-28-oic acid (d), corosolic acid (e) (Figure 1) [8].

### 2.3. Cell Culture 

Hepatoma H22 cells were purchased from Chinese Academy of Sciences (Shanghai, China), preserved in the Tumor Research Institute of TCM Department, Changhai Hospital, SMMU (Shanghai, China), grown in RPMI-1640 medium supplemented with 10% FBS, 100 IU/mL penicillin and 100 *μ*g/mL streptomycin at 37°C in 5% CO^2^ in a humidified incubator.

### 2.4. Animals

 Kunming mice weighing 18–22 g, purchased from Animal Center, SMMU, received humane care according to the criteria outlined in the “Guide for the Care and Use of Laboratory Animals” published by the National Institute of Health (NIH publication 86–23 revised 1985). 

### 2.5. Transplanted Hypodermal Hepatoma H22-Bearing Mice

We observed the cell morphology in the microscope, quantitated, and suspended cell density to about 2 × 10^7^ cells/mL. About 0.2 mL cell suspensions were then subcutaneously inoculated into the left oxter region of each mouse [6]. Calculate mice weight and tumor size each other day. When the tumor size reached an average diameter of 5-6 mm (about 5 days later), tumor-bearing mice were divided into 3 groups randomly, each group with 10 mice. (1) High-TSAVD: mice in this group were injected with 0.2 mL TSAVD in abdominal cavity with a concentration of 1 g/kg/d for 10 days, TSAVD was dissolved completely in DMSO, and dissolved in distilled water containing 0.5% tween-80 and 0.5% carboxymethylcellulose. (2) Low-TSAVD group: mice in this group were injected with TSAVD at half concentration of first group. (3) Control group: mice in this group were treated with solventia at concentration of the first group. 

### 2.6. Evaluation of Antitumor Effect *In Vivo*

Therapeutic response was evaluated by mouse activities, body weights, and tumor growth. The long (a) and short (b) diameters of the tumors were measured with a slide caliper each other day after treatment. Tumor size was calculated as follows: volume  = (*a* × *b* ^2^)/2. Mice were sacrificed and peeled next day after injection, and tumors were weighted. Inhibitive ratio (IR) was calculated as follows: IR = (1  −  average tumor weight of treated group/control group)  ×  100%. 

The fixed tumor tissues were progressively dehydrated in solutions containing an increasing percentage of ethanol embedded in paraffin, sectioned at  5 *μ*m thickness, deparaffinized, and stained with hematoxylin and eosin. Immunohistochemical staining for CD34 was performed by the immunoperoxidase technique. Microvessel density (MVD) was determined in the five most intense vascularization areas (hotspots) of each section by observing at 200x magnification. All discrete clusters or single cells stained positive for CD34 were counted as one vessel. The average count was regarded as MVD for each case. IQAS medical image quantitative analysis system was used to know vascular endothelial growth factor (VEGF) and basic fibroblast growth factor (bFGF) expression rate. VEGF, bFGF-positive expression rates were determined by brown spot in the microscope visual field [9]. Three visions were selected randomly in each specimen at high magnification. The spleen, thymus, kidney, liver, and lung were also observed by naked eye to see whether they had adverse reactions, while pathochanges of the organs were also viewed in microscope. Evaluation criterion: the therapy was inefficient when IR was less than 30%, efficient when IR was more than 30%. 

### 2.7. Experimental Pulmonary Metastasis Hepatoma H22 Bearing Mice

In this experiment, we quantitated the density of hepatoma H22 cells to about 2.5 × 10^7^ cells/mL, injected each mouse by vena caudalis with 0.2 mL. Thirty mice were divided into 3 groups randomly the next day. Each mouse was treated in the same way of the former experiment for 2 weeks. The mice were weighted every other day and activities were observed in treatment. They were sacrificed the next day after the last injection. Metastatic nodes number, distribution, and size in lung were observed by naked eye and microscope, pathochanges of lung tissues were detected by hematoxylin and eosin stain [10]. The metastasis inhibitory rate was calculated as follows: IR = (1  − average  number of lung metastatic nodes in treated group/average number of lung metastatic nodes in control group)  ×  100%. The weight of spleen was read in electronic scale, the spleen index was calculated to see whether it had vice effects on immune system. The white blood cells, red blood cells, and platelets were also observed in the three groups to test whether it had vice effects on blood system. 

### 2.8. Statistical Analysis

All data were processed by SPSS, and results were expressed as mean ± standard deviation. The statistical significance of differences among different groups was determined with a 95% confidence interval by the ANOVA test for normally distributed data, or the Kruskal-Wallis *H* Test, Fridman Test for nonnormally distributed data. 

## 3. Results 

### 3.1. HCC Growth Inhibition *In Vivo*

**Figure 2:**
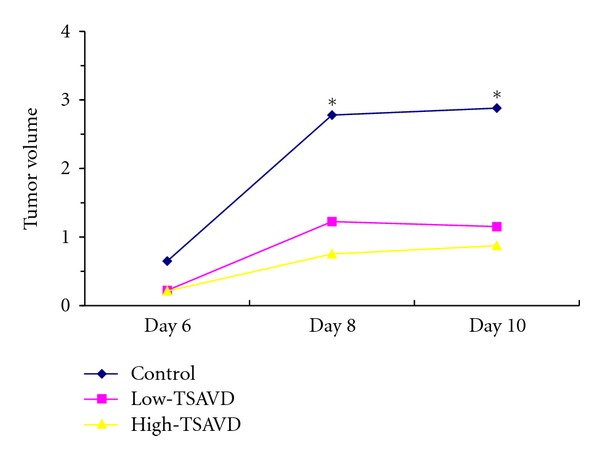
Tumor volume of transplanted hypodermal hepatoma H22 bearing mice in 3 groups.

**Figure 3:**
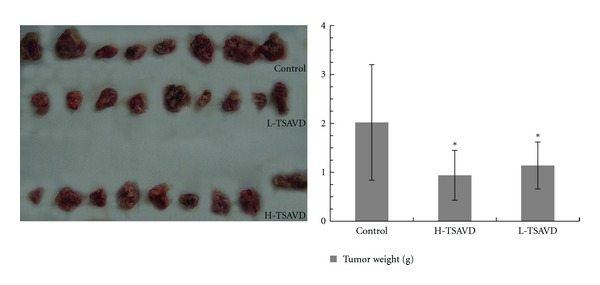
Anti-growth effect of TSAVD on transplanted hypodermal hepatoma H22 bearing mice (*P < 0.05 versus control group).

From the experiments, we could see that tumor in TSAVD-treated group was smaller than that of control group as shown in Figures 2 and 3. The tumor weight in TSAVD-treated groups was obviously less than that of control group at the 8th and 10th day. The tumor weight in TSAVD-treated group was less than that of control group, and the difference showed a statistical significance. IRs were 53.2% of H-TSAVD group, 43.5% of L-TSAVD group. No metastasis tumors and changes were found in the other organs such as lung, liver, and kidney. The spleen, thymus indexes in three groups show no differences.

**Figure 4:**
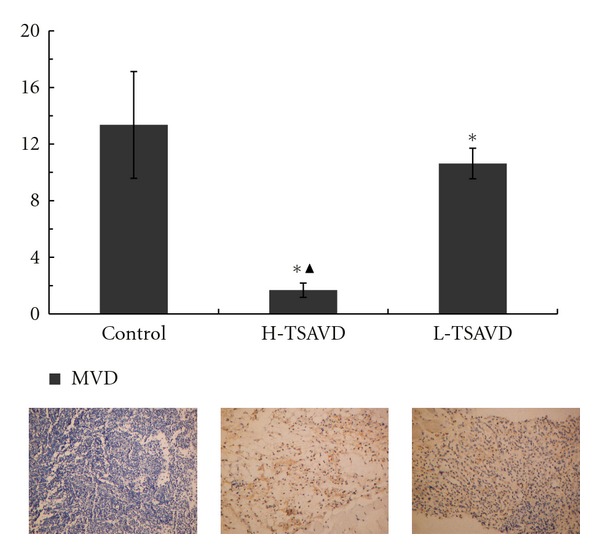
CD34 expressions of transplanted hypodermal hepatoma H22 bearing mice (*P < 0.05 versus control group, ^▲^ *P *< 0.05 versus H-TSAVD group).

As Figure 4 shows, in our experiments, the expressions of MVD within the three groups were different (*P* < 0.05): about (1.68 ± 0.50), (10.63 ± 1.08), and (13.36 ± 3.77) in H-TSAVD, L-TASVD group and control group. Expressions of VEGF in H-TSAVD were 1.6, different from control group (*P* < 0.05), while no difference was shown in H-TSAVD and L-TSAVD groups. The expressions of bFGF within the three group were different (*P* < 0.05), about 3.4, 3.1, and 4 in H-TSAVD, L-TASVD group, and control group. 

### 3.2. HCC Metastasis Inhibition *In Vivo*

**Figure 5:**
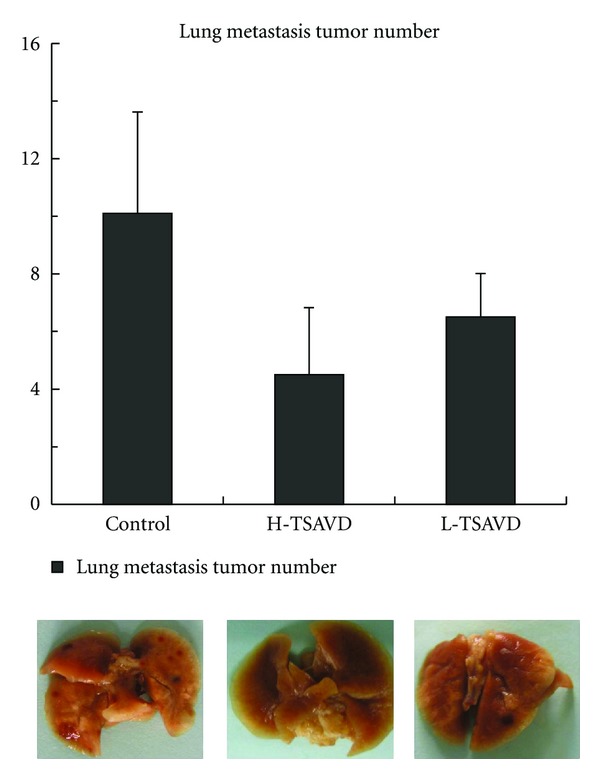
Antimetastasis effect of TSAVD on pulmonary metastasis hepatoma H22 bearing mice (*P < 0.05 versus control group).

 No difference was shown in the weights of mice in three groups, but the numbers of pulmonary metastases tumor were different in H-TSAVD, L-TSAVD, and control groups, which were (4.5 ± 2.33), (6.5 ± 1.51), and (10.1 ± 3.52), respectively, as shown in Figure 5. The number in TSAVD-treated groups was significantly less than that of control group (*P* < 0.01), but no difference was shown between H-TSAVD and L-TSAVD groups. IRs were 55.49% and 35.71% in H-TSAVD, L-TSAVD groups. 

**Figure 6:**
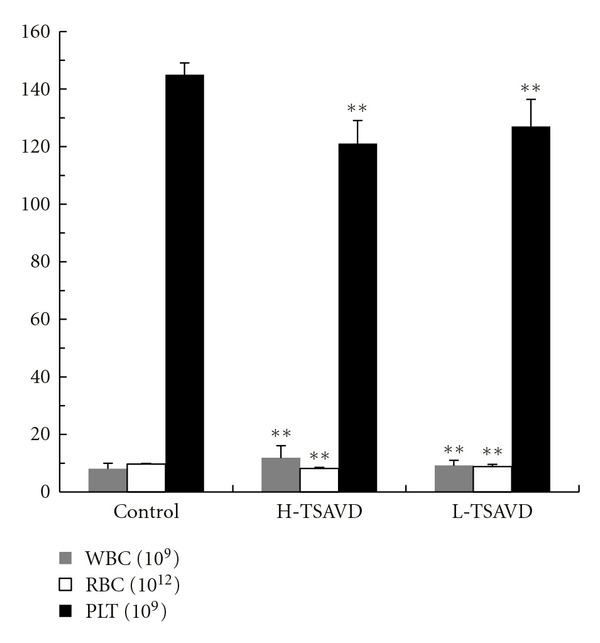
Blood cells of pulmonary metastasis hepatoma H22 bearing mice (**P < 0.01 versus control group).

**Figure 7:**
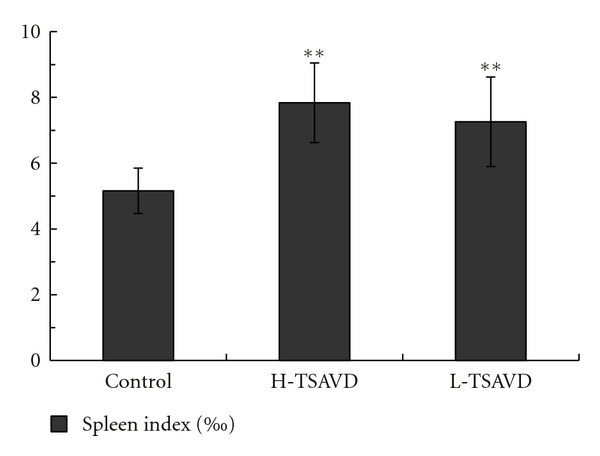
Spleen index of pulmonary metastasis hepatoma H22 bearing mice (**P < 0.01 versus control group).

The white blood cells, red blood cells, and platelets were different between TSAVD-treated group and control group (*P <* 0.01), as shown in Figure 6, but no difference was shown in H-TSAVD, L-TSAVD groups (*P >* 0.05). The spleen index of the three groups showed statistic significance as Figure 7 show (*P <* 0.01).

## 4. Discussion 

Overgrowth and metastasis are the two major characteristics of malignancy, so researches of effective Chinese herbs used for cancer therapy mainly focus on affecting each progress of carcinoma, involving precancerous lesion prevention, anti-tumor, antimetastasis, and so forth [11]. In solid tumors, angiogenesis is well characterized as a critical step for growth, invasion, and metastasis [12]. Angiogenesis refers to the process of new blood vessel formation from a pre-existing vasculature which occurs in either physiological or pathological conditions [13]. This process is tightly regulated by a series of pro- and antiangiogenic molecules in normal physiology; however, serious consequences may arise when this equilibrium is broken [14]. Tumor angiogenesis shows a markedly increasing proliferation of endothelial cell and has significant functional and structural differences in the vascular plexus. Tumor cells promote vessel formation through the expression of angiogenic molecules or their induction in the microenvironment [15, 16].

Among the proangiogenic molecules, vascular endothelial growth factor (VEGF) and basic fibroblast growth factor (bFGF) have been identified to drive tumor-related angiogenesis. VEGF is the best-characterized angiogenic cytokine and the most potent angiogenesis inducer [2]. The crucial regulators of the angiogenesis process associated with tumor development and metastasis are VEGF and their receptors [17]. Hepatoma H22 tumor cells with significant invasion and metastasis can propagate easily in mice, so they are extensively applied for basal investigation and pharmic screening. Also in this study, transplanted hypodermal and experimental pulmonary metastasis hepatoma H22-bearing mice were used. 

*Actinidia valvata* Dunn was widely used for hepatoma therapy by traditional Chinese doctor in clinical practice, exhibited antitumor and anti-inflammatory effects, and it had been used widely for hepatoma, lung carcinoma, and myeloma therapy for a long time. It is abundant in amino acids and inorganic elements that people need. Antitumor effects of *Actinidia valvata* Dunn had been investigated for the past five years, its injection had been confirmed to have a significant antitumor effect, significantly restrain the growth of primarily hypodermal tumor with no cell-mediate immunity inhibitive effects *in vivo* [4, 5]. TSAVD was isolated for the first time from the root of *Actinidia valvata* Dunn. In previous studies, we have found that TSAVD has inhibitory effects on HCC metastasis *in vitro*. The present study aimed to extend the previous study of TSAVD and to evaluate its anti-HCC mechanism *in vivo*. 

Experimental data presented here showed that TSAVD played the potent antiangiogenic activity in mice *in vivo*. The antiangiogenic mechanism of TSAVD lies in the ability to inhibit hepatoma tumor growth and migration, the results showed that TSAVD can effectively restrain the growth and metastasis of hepatoma H22 *in vivo*.

According to the volume and immunohistochemistry of the tumors, the TSAVD-treated group showed a lower tumor volume and CD34 expression level (a lower blood vessel density). The result that TSAVD decreased the tumor volume and MVD implied TSAVD had the action of antitumor and antiangiogenesis activity. The expressions of bFGF in TSAVD-treated groups were less than control group, and also in H-TSAVD group, the expressions were less than that of L-TSAVD group. Expressions of VEGF in H-TSAVD were different from other two groups. In the process of tumor growth, the two-way paracrine action between tumor cells and vascular endothelial cells leads to obvious increase of tumor blood vessels and promote the tumor growth.

The pathochanges of metastasis tumor also show that TSAVD can accelerate putrescence of tumor in pulmonary metastasis hepatoma H22-bearing mouse experiments, Results also showed that TSAVD may improve levels of WBC, RBC, and PLT in mice, having no restraint effects like most chemotherapy medicine. 

To sum up, the present study demonstrates that TSAVD can restrain growth and metastasis of HCC with no vice blood effects *in vivo*, restrain angiogenesis maybe one of the mechanism. Further studies will be done in our laboratory to konw the effects of TSAVD on other tumors. 

## References

[1] Wu MC (2003). Traditional Chinese medicine in prevention and treatment of liver cancer: function, status and existed problems. *Zhong Xi Yi Jie He Xue Bao*.

[2] Tan WF, Lin LP, Li MH (2003). Quercetin, a dietary-derived flavonoid, possesses antiangiogenic potential. *European Journal of Pharmacology*.

[3] Ribatti D, Nico B, Morbidelli L (2001). Cell-mediated delivery of fibroblast growth factor-2 and vascular endothelial growth factor onto the chick chorioallantoic membrane: endothelial fenestration and angiogenesis. *Journal of Vascular Research*.

[4] Wan XY, Zhang YN, Zhang C (2004). Experimental study on anti-hepatocarcinoma effect of maorenshen injection *in vitro*. *Journal of Zhejiang College of Traditional Chinese Medicine*.

[5] Zhang YN, Liu L, Ling CQ (2006). Inhibition effect of active fraction from *Actinidia valvata* on growth of transplanated mouse tumor cells and preliminary study of its mechanism. *China Journal of Chinese Materia Mediea*.

[6] Wan XY, Zhang C, Ling CQ, Li B (2004). Anti-hepatocarcinoma activity and immunopharmacological effects of Maorenshen injection. *Journal of Zhejiang College of Traditional Chinese Medicine*.

[7] Zheng GY, Xin HL, Li B, Xu YF, Yi TJ, Ling CQ (2012). Total saponin from root of *Actinidia valvata* Dunn prevents the metastasis of human hepatocellular carcinoma cells. *Chinese Journal of Integrative Medicine*.

[8] Xin HL, Yue XQ, Xu YF (2008). Two new polyoxygenated triterpenoids from *Actinidia valvata*. *Helvetica Chimica Acta*.

[9] Mikami S, Oya M, Mizuno R, Murai M, Mukai M, Okada Y (2006). Expression of Ets-1 in human clear cell renal cell carcinomas: implications for angiogenesis. *Cancer Science*.

[10] Du B, Wang SY, Shi XF, Zhang CF, Zhang ZZ (2011). The effect of 2-methoxyestradiol liposome on growth inhibition, angiogenesis and expression of VEGF and Ki67 in mice bearing H22 hepatocellular carcinoma. *Tumori*.

[11] Ling CQ (2003). Problems in cancer treatment and major research of integrative medicine. *Zhong Xi Yi Jie He Xue Bao*.

[12] Liu S, Yu M, He Y (2008). Melittin prevents liver cancer cell metastasis through inhibition of the Rac1-dependent pathway. *Hepatology*.

[13] Carmeliet P (2003). Angiogenesis in health and disease. *Nature Medicine*.

[14] Lee H, Baek S, Joe SJ, Pyo SN (2006). Modulation of IFN-*γ* production by TNF-*α* in macrophages from the tumor environment: significance as an angiogenic switch. *International Immunopharmacology*.

[15] Martínez A (2006). A new family of angiogenic factors. *Cancer Letters*.

[16] Jakob C, Sterz J, Zavrski I (2006). Angiogenesis in multiple myeloma. *European Journal of Cancer*.

[17] Brychtova S, Fiuraskova M, Malikova J (2006). 94 POSTER Angiogenesis in human cutaneous tumors. *European Journal of Cancer Supplements*.

